# PPAR-γ in the Cardiovascular System

**DOI:** 10.1155/2008/745804

**Published:** 2007-12-06

**Authors:** Sheng Zhong Duan, Christine Y. Ivashchenko, Michael G. Usher, Richard M. Mortensen

**Affiliations:** ^1^Department of Molecular and Integrative Physiology, University of Michigan Medical School, Ann Arbor, MI 48109, USA; ^2^Department of Pharmacology, University of Michigan Medical School, Ann Arbor, MI 48109, USA; ^3^Department of Internal Medicine, Metabolism Endocrinology and Diabetes Division, University of Michigan Medical School, Ann Arbor, MI 48109, USA

## Abstract

Peroxisome proliferator-activated receptor-γ (PPAR-γ), an essential transcriptional mediator of adipogenesis, lipid metabolism, insulin sensitivity, and glucose homeostasis, is increasingly recognized as a key player in inflammatory cells and in cardiovascular diseases (CVD) such as hypertension, cardiac hypertrophy, congestive heart failure, and atherosclerosis. PPAR-γ agonists, the thiazolidinediones (TZDs), increase insulin sensitivity, lower blood glucose, decrease circulating free fatty acids and triglycerides, lower blood pressure, reduce inflammatory markers, and reduce atherosclerosis in insulin-resistant patients and animal models. Human genetic studies on PPAR-γ have revealed that functional changes in this nuclear receptor are associated with CVD. Recent controversial clinical studies raise the question of deleterious action of PPAR-γ agonists on the cardiovascular system. These complex interactions of metabolic responsive factors and cardiovascular disease promise to be important areas of focus for the future.

## 1. INTRODUCTION

Cardiovascular disease (CVD) is the major cause of morbidity and mortality in developed countries [1]. Searching for the underlying risk factors has revealed that a cluster of contributors is often present simultaneously. This risk factor clustering, most notably the core trio of insulin resistance, dyslipidemia, and hypertension, has been called by a number of different names including metabolic syndrome (MetS), insulin resistance syndrome, the deadly quartet, and Syndrome X [1–7]. Although somewhat controversial, the usefulness of clustering this syndrome remains clear. The mechanistic connections among the trio are not completely understood.

A major focus is to understand the biological and molecular mechanisms underlying this syndrome and to develop better treatment. One class of molecules that are proposed to be important in the etiology of MetS is the nutrient-sensing nuclear transcription factors, peroxisome proliferator-activated receptors (PPARs), and the related liver X receptors (LXRs) [7]. Among these nuclear receptors, PPAR-*γ* is of intense interest, not only because its ligands, thiazolidinediones (TZDs), are clinically used for T2DM, but also because it may be a nexus that connects metabolic disorders to CVD [1, 4–9]. In addition to its important roles in insulin sensitivity and glucose homeostasis, PPAR-*γ* is also associated with CVD such as coronary heart disease, atherosclerosis, and stroke [7, 10]. MetS is also linked to cardiac hypertrophy because populations with MetS have higher prevalence of cardiac hypertrophy [11–14]. However, action of PPAR-*γ* agonists is not only of metabolism in insulin responsive tissues, but also more directly in the inflammatory, cardiac, and vascular cells. The components of MetS are common risk factors to CVD [1]. In this review, we will focus on PPAR-*γ* in the cardiovascular (CV) system, including its expression, gain, and loss of function, and the mechanisms by which it functions in cardiovascular cells.

## 2. PPAR-γ GENE AND ITS EXPRESSION OF CV-RELEVANT TISSUES

PPAR-*γ* is the most extensively studied PPAR, even though the cloning of this receptor came four years later than that of PPAR-*α* [15]. The PPAR-*γ* gene extends over more than 100 kb of genomic DNA. It includes six common coding exons: one exon for the N-terminal A/B domain, two exons for the DNA binding domain, with each one encoding one of the two zinc fingers, one exon for the hinge region, and two exons for the ligand binding domain in the C-terminal region [16, 17]. There are two major splice isoforms in the mouse, PPAR-*γ*1 and PPAR-*γ*2, whereas at least two other isoforms, PPAR-*γ*3 and PPAR-*γ*4, have also been identified in other species including humans [16]. PPAR-*γ*1 is encoded by eight exons, comprising two *γ*1-specific exons, A1 and A2, that constitute the $5^{\prime }$-untranslated region, and the six coding exons that are common to both *γ*1 and *γ*2 mRNAs. The PPAR-*γ*1 protein consists of 477 amino acids [16]. The PPAR-*γ*2 mRNA is composed of seven exons, the additional one, exon B, comprising the *γ*2 $5^{\prime }$-untranslated region and an additional N-terminal amino acid sequence specific for *γ*2. As a result, PPAR-*γ*2 is a larger protein, consisting of 505 amino acids [16].

The function PPAR-*γ* was initially recognized in adipose tissue [18], although its expression was first identified in other tissues [15]. It is well expressed in cardiovascular-system-relevant tissues such as heart, endothelium, vascular smooth muscle, kidney, and macrophages [10, 19–23]. PPAR-*γ*2 is mainly expressed in adipocytes while PPAR-*γ*1 is more widely expressed [23].

## 3. PPAR-γ LIGANDS

**Natural ligands**

Several polyunsaturated fatty acids and their metabolites have been identified as PPAR-*γ* ligands although no ligand has clearly been identified as a critical physiologic ligand. Endogenous ligands including 15-deoxy-Δ^12,14^ prostaglandin J2 (15-d-PGJ2) [24]. 15-d-PGJ2 had been one of the most promising candidates for the endogenous PPAR-*γ* ligand. It binds to PPAR-*γ* with a dissociation constant (*K*_d_) in the low-micromolar range and can activate PPAR-*γ* target genes at concentrations at or near the *K*_d_ [25]. However, it has never been definitively proven to exist in vivo, nor are its effects that are specific to PPAR-*γ* [25]. Other natural ligands of PPAR-*γ* include 9- and 13-hydroxyoctadecadienoic acid (HODE), which are components of oxidized low-density lipoprotein [26], and 12- and 15-hydroxyeicosatetraenoic acid (HETE) [27]. Some researchers have argued that fatty acids are the important natural ligands although they are fairly low affinity ligands. A recent class of high-affinity ligands, the nitrolipids, has been identified, but their physiologic function and the role of PPAR-*γ* in their effects have not yet been fully delineated [28].

**Synthetic ligands**

TZDs, or “glitazones,” are a class of pharmaceutical compounds used clinically as insulin sensitizers in patients with T2DM [17]. The first clinically used agent in this class, troglitazone (Rezulin), was removed from the market because of rare but life-threatening hepatic toxicity. Fortunately, its successors, rosiglitazone (Avandia), and pioglitazone (Actos), have not been linked to this side effect [23]. More than 15 million prescriptions for these TZDs are dispensed annually in the United States alone. However, adverse effects such as edema and weight gain have been problematic [23]. Another side effect of TZDs in animals is cardiac hypertrophy, which has limited the approved doses of these drugs for clinical use [29–33]. More recently, increased risks of myocardial infarction and possibly death from cardiovascular causes have been reported to be associated with rosiglitazone (Avandia) treatment [34], although the result is controversial [35].

**TZDs' “on-target” and “off-target” effects**

Compelling evidence has shown that PPAR-*γ* is the main target of TZDs. PPAR-*γ* mediates the insulin sensitizing effects of TZDs in fat, skeletal muscle, and liver [36–39]. TZDs' effects on fluid retention and weight gain are also dependent on PPAR-*γ* [40, 41]. However, several studies demonstrate that some of TZDs' effects are independent of PPAR-*γ* or “off-target.” In macrophages, it has been recognized that although TZDs modulate lipid metabolism through PPAR-*γ*, some of TZDs' anti-inflammatory effects are independent of it [42] although only at higher doses. Some of the antiproliferative effects of TZDs in embryonic stem cells [43] or cancer cell lines [44, 45] are independent of PPAR-*γ*. Further, PPAR-*γ* in skeletal muscle and liver may not be mediating TZDs' insulin sensitizing effects under different conditions or in different models [38, 39, 46]. Loss-of-function studies have provided additional insight on the possible “off-target” effects of TZDs.

Understanding which TZD effects are PPAR-*γ* independent is an important issue for designing more specific PPAR-*γ* agonists with fewer side effects. TZDs induce cardiac hypertrophy in animals [29–33] independent of cardiac PPAR-*γ* [47]. TZDs also increase the incidence of congestive heart failure [48] presumably due to fluid retention caused by PPAR-*γ* activation in the kidney [40, 41]. Myocardial infarction incidence is increased in meta-analysis of clinical trials [34], but it is not known whether this side effect is mediated by PPAR-*γ* or whether this finding will be confirmed in a prospective study [35]. Further, defining the role of PPAR-*γ* in these effects would provide guidance for the design of the next generation of TZDs.

## 4. GAIN AND LOSS OF PPAR-γ FUNCTION IN THE CV SYSTEM

Although originally found to be critical in adipogenesis and regulating insulin signaling, PPAR-*γ* is also important in CV system [16, 17, 49, 50]. Human genetic studies have revealed that PPAR-*γ* mutation in humans can result in either gain-of-function or loss-of-function [51]. In animals, gain-of-function studies of PPAR-*γ* have mostly utilized agonists, particularly synthetic ones (TZDs); Loss-of-function studies have used knockdown or knockout and transgenic mouse models of mutant PPAR-*γ*, which are powerful tools to study physiological mechanisms. The outcome of these approaches in studying PPAR-*γ* in CV system has been fruitful and sometimes surprising.

**Human mutations**

Pro12Ala mutation is a loss-of-function mutation and has been reported to be associated with not only increased protection against insulin resistance and type-2 diabetes [52–56], but also a decreased incidence of myocardial infraction [57] and lower diastolic blood pressure [58]. These cardiovascular effects are likely independent of metabolic impact of this mutation [57, 58].

Pro467Leu, Val290Met, Phe388Leu, and Arg425Cys are all loss-of-function mutations (dominant negative) and have been associated with partial lipodystrophy, insulin resistance, diabetes, and hypertension [59–62], although it is not known whether the elevated blood pressure is due to impaired insulin sensitivity.

C161T mutation is a silent polymorphism and has been reported to be associated with reduction in coronary artery disease, likely independent of obesity and of lipid abnormalities, possibly through direct effects on local vascular wall, implicating the protective role of PPAR-*γ* in atherogenesis [63].

However, ligand binding domain mutants of PPAR-*γ* with dominant negative actions have been shown to be promiscuous, stimulate associations with nuclear receptor corepressor (N-CoR) and silencing mediator of retinoid and thyroid receptors (SMRT), and inhibit activities of all three wild-type PPARs [64]. These less specific properties of the mutants need to be explored in the human mutations to determine which effects on metabolic syndrome are mediated through PPAR-*γ* or the PPARs, PPAR-*α* or PPAR-*δ*.

**Germline gene inactivation**

Homozygous germline PPAR-*γ* knockout mice die at E10 due to defects in trophoblast [65, 66]. Heterozygous mutations are viable, have less adipose tissue, and are more insulin sensitive than wild-type counterparts [67, 68]. This illustrates the complex response to gene dosage of PPAR-*γ*.

**PPAR-*γ* and hypertension**

PPAR-*γ* agonists can lower blood pressure and this effect may be at least partially independent of their insulin-sensitizing effects [17, 49, 50]. Dominant negative mutation of PPAR-*γ* (Pro467Leu) in mice results in hypertension and fat redistribution but not insulin resistance or diabetes seen in the same mutation in humans [69]. Another line of dominant negative PPAR-*γ* mutant mice (Leu466Ala) have hypertension (female only) and insulin resistance [70]. As discussed above, dominant negative PPAR-*γ* mutants can be more promiscuous, inhibiting activity of other PPARs, so we cannot at this point conclude that these mutants strictly act by altering only PPAR-*γ* activity [64]. Therefore, comparison to loss of function mutants and knockouts is critical.

Embryonic lethality of the germline PPAR-*γ* knockout has been rescued by breeding Mox2-Cre, in which Cre recombinase is expressed in epiblast-derived tissue but not other tissues, to floxed PPAR-*γ* mice [71]. The generalized PPAR-*γ* knockout mice have lipodystrophy and insulin resistance as expected. Surprisingly, they have hypotension rather than hypertension. This is paradoxical because PPAR-*γ* agonists lower blood pressure [72–74]. Knockout and agonist having the same phenotype may be resolved by testing the hypothesis that PPAR-*γ* suppresses certain key gene expression to control blood pressure and that both agonist and knockout can relieve the suppression. Further, the phenotypes in the generalized PPAR-*γ* knockout mice suggest that hypertension is separable from lipodystrophy or insulin resistance, even though they are highly associated in humans [59, 61] and in A-ZIP mice [71]. Mechanistically, the vasculature from these generalized PPAR-*γ* knockout mice has defects in both relaxation and contraction, contributing to the hypotension.

It has not been completely determined whether the hypotension phenotype seen in the generalized PPAR-*γ* knockout mice is attributable to PPAR-*γ* deficiency in vascular endothelium or smooth muscle or both. Endothelium-specific PPAR-*γ* knockout mice are reported to be not having any phenotype at baseline, although this study only used tail cuff to measure the blood pressure [75]. When fed with high fat diet, they have higher blood pressure and heart rate than their wild type control mice. Rosiglitazone does not affect the diet-induced hypertension in these knockout mice, although the decrease of blood pressure typically seen in treated wild-type control mice was not reported [75]. Smooth muscle-specific PPAR-*γ* knockout mouse model may clarify the role of smooth muscle PPAR-*γ* in blood pressure regulation.

The kidney is an important organ in controlling blood pressure and PPAR-*γ* is expressed in this organ, although PPAR-*γ* deficiency in collecting duct does not alter blood pressure in mice [40, 41]. The knockout does block weight gain, fluid retention, and blood volume expansion caused by TZDs [40, 41]. These results indicate that PPAR-*γ* deficiency in collecting duct is unlikely to contribute to the hypotension phenotype seen in the generalized PPAR-*γ* knockout mice [71].

**PPAR-*γ* and cardiac hypertrophy**

PPAR-*γ* agonists have been shown to inhibit hypertrophy of cultured neonatal rat ventricular cardiomyocytes induced by mechanical stress or angiotensin II, and cardiac hypertrophy induced by aortic constriction in rats and mice [76–78]. The inhibition on hypertrophy was accompanied by the inhibition on expression of embryonic genes, including atrial natriuretic peptide (ANP), brain natriuretic peptide (BNP), skeletal *α*-Actin, as well as that of endothelin-1 that can induce cardiac hypertrophy [76–78]. Aortic constriction causes more profound cardiac hypertrophy in heterozygous PPAR-*γ* knockout mice than wild-type controls, further indicating the involvement of PPAR-*γ* in cardiac growth [77]. Nuclear Factor-Kappa Beta (NF-*κ*B) pathway is at least partially mediating the inhibition on hypertrophic growth in vitro [76]. More recently, pioglitazone has been reported to inhibit the gene expression of inflammatory cytokines such as interleukin (IL)-1*β*, IL-6 [79], suggesting the possible involvement of PPAR-*γ*'s anti-inflammatory activity in cardiac hypertrophy. Paradoxically, TZDs also induce cardiac hypertrophy in mice, rats, and dogs [30–33].

The cardiomyocyte-specific PPAR-*γ* knockout mice have age-progressive cardiac hypertrophy with preserved systolic cardiac function [47]. Rosiglitazone induce cardiac hypertrophy in both knockout mice and wild-type littermate control mice, demonstrating that this TZD's hypertrophic effect is at least partially independent of PPAR-*γ* in cardiomyocytes [47]. Whether the cardiac hypertrophy caused by rosiglitazone occurs through PPAR-*γ* independent effects in cardiomyocytes, PPAR-*γ* in nonmyocyte cells, or blood volume expansion [33, 48], remains to be further determined. Studying these knockout mice under pathological stimulations such as pressure overload may yield more meaningful results to understand the function of PPAR-*γ* in the heart.

Rosiglitazone and cardiomyocyte-specific PPAR-*γ* knockout activate distinctly different hypertrophic pathways [47]. Rosiglitazone increases phosphorylation of both p38 mitogen-activated protein kinase (p38-MAPK) and extracellular signal-related kinase (ERK) 1/2 in the heart. The activation of p38-MAPK is independent of cardiomyocyte PPAR-*γ* and that of ERK1/2 is dependent. The activation of either ERKs or p38-MAPK is sufficient to induce hypertrophy [80, 81] and may therefore contribute to the cardiac hypertrophy induced by rosiglitazone.

The cardiomyocyte-specific PPAR-*γ* knockout mouse hearts were found to have increased expression of cardiac embryonic genes ANP and *β*-myosin heavy chain (*β*-MHC), and elevated NF-*κ*B activity. Embryonic gene expression in adult hearts is a one of the characteristics of pathological cardiac hypertrophy [82, 83]. NF-*κ*B is both necessary and sufficient for hypertrophic growth of cardiomyocytes [84]. Therefore, NF-*κ*B activation is likely to be part of the mechanisms that PPAR-*γ* deficiency in cardiomyocytes induces cardiac hypertrophy. However, the interaction between these two transcription factors needs to be further characterized.

Another cardiomyocyte-specific PPAR-*γ* knockout mouse line also has progressive cardiac hypertrophy and accompanied elevation of cardiac gene expression (ANP and skeletal *α*-actin) [85]. However, these mice also have dilated cardiomyopathy, heart failure, and mitochondrial oxidative damage. Increased myocardial superoxide content, instead of NF-*κ*B activation, seems to be mediating the severe cardiac phenotype [85]. Similarly, when different floxed PPAR-*γ* mice were used to delete this receptor in skeletal muscle specifically, one mouse line [37] had more severe phenotypes than the other [46]. It is not clear whether these phenotypic differences are because of the different genetic design for the deletion or purely genetic background differences.

**PPAR-*γ*, inflammation, and atherosclerosis**

PPAR-*γ* is not only expressed in macrophages, endothelial cells, and smooth muscle cells in normal vasculature [10, 19–23], but also in atherosclerotic lesions [86, 87]. PPAR-*γ* agonists reduce atherosclerosis in human patients and animal models [88–92] even though there were concerns that these compounds could be proatherogenic because they may promote the macrophages uptake of lipids and speed the foam cell formation [26]. These antiatherogenic effects can be independent of their beneficial effects on metabolism [93, 94]. More direct antiatherogenic function of PPAR-*γ* was demonstrated by the result that transplantation of PPAR-*γ* deficient bone marrow to low-density lipoprotein (LDL) receptor null mice led to a significant increase in atherosclerosis [95]. The beneficial effects are largely attributable to PPAR-*γ*' s anti-inflammation activity and its role in modulating lipid homeostasis in macrophages.

Vascular inflammation has been increasingly appreciated as an important factor in the pathogenesis of atherosclerosis [96–99]. The importance of macrophage PPAR-*γ* in CVD has begun to be appreciated since foam cells in atherosclerotic lesions were found to have high level of PPAR-*γ* expression [86, 87]. PPAR-*γ* activation decreases inflammatory cytokines (e.g., tumor necrosis factor-*α*, IL-6, and IL-1*β*) produced by macrophages [100, 101]. By inducing the expression of LXR-*α* and ATP-binding cassette A1, PPAR-*γ* activation promotes cholesterol efflux from macrophages resulting in inhibition of foam cell formation [95]. Consistently, macrophage-specific PPAR-*γ* knockout mice have reduced basal cholesterol efflux, most likely because of decreased expression of lipoprotein lipase, scavenger receptor CD36, LXR-*α*, and ATP-binding cassette G1 [21]. More profound effects on macrophages by PPAR-*γ* are also possible since it has been recently shown that PPAR-*γ* controls alternative activation of macrophages and can thereby improve insulin resistance [102]. It is likely that this effect on differentiation of macrophages is also important in effects of CVD.

Endothelial cells play a key role in the inflammatory process of vasculature responding to injuries [96–99]. TZDs have been shown to reduce superoxide generation and inhibit the expression of vascular cell adhesion molecule-1, intercellular cell adhesion molecule-1, and lectinlike oxidized LDL receptor, and hence inhibit inflammation in endothelial cells [103–106], suggesting an important role of endothelial PPAR-*γ* in the development of atherosclerosis. The existing endothelium-specific PPAR-*γ* knockout mice [75] and generalized PPAR-*γ* knockout mice [71] can be useful tools to study the function of endothelial PPAR-*γ* in atherosclerosis.

Different cell types are reported to have different mechanisms for PPAR-*γ* to inhibit inflammation. In intestinal Caco-2 cells, PPAR-*γ* inhibits inflammation by binding to NF-*κ*B and facilitating its nuclear export [107]. In macrophages, PPAR-*γ* prevents corepressor complex N-CoR dissociation from the promoters of NF-*κ*B responsive inflammatory gene inducible nitric oxide synthase and therefore represses its expression [108]. It remains to be determined whether PPAR-*γ* in endothelial or other vascular cells uses one of these pathways or an entirely different mechanism.

The growth and movement of vascular smooth muscle cell within neointima is one of the key steps leading to the formation of atherosclerotic plaque [96]. PPAR-*γ* agonists have been shown to block the proliferation and increase the apoptosis of vascular smooth muscle cells, suggesting more beneficial effects of PPAR-*γ* activation in vasculature [109, 110].

**PPAR-*γ* and cardiac remodeling**

Cardiac remodeling after ischemic injury is one of the major causes that lead to heart failure [111, 112]. The remodeling process is characterized by myocyte hypertrophy and cardiac fibrosis [111, 112]. PPAR-*γ* agonists attenuate this remodeling process after ischemia in experimental animals [113]. Recent in vitro studies on PPAR-*γ* in cardiac fibroblasts, a major source of fibrillar collagens that lead to fibrosis [111, 112], have revealed more mechanistic insights.

Pioglitazone reduces cell growth, synthesis of collagen type I, and expression of matrix metalloproteinase-1 in cardiac fibroblasts undergone anoxia-reoxygenation or treated with angiotensin II, likely through inhibition of reactive oxygen species generation and NF-*κ*B activation [114, 115]. Brain natriuretic peptide has been implicated in these effects [116]. In cultured cardiac fibroblasts, PPAR-*γ* agonists induce the expression of vascular endothelial growth factor, a crucial player in the infarcted/ischemic heart, further indicating the beneficial effects of PPAR-*γ* agonists in cardiac remodeling [117]. However, all of these studies are based upon gain-of-function results. Further investigation using loss-of-function studies would advance our understanding the role of PPAR-*γ* in cardiac fibroblasts and cardiac remodeling and provide more therapeutic guidance.

**PPAR-*γ* in cardiovascular side effects of TZDs**

Despite the obviously beneficial effects that TZDs have in CV system [118], these compounds have some cardiovascular side effects that are dangerous to be overlooked. As mentioned above, TZDs induce cardiac hypertrophy in animals, a limitation to the dosages in their clinic use.

Congestive heart failure remains to be one of the major contraindications to the clinical use of TZDs [48]. This is presumed to be secondary to the fluid retention caused by activation of PPAR-*γ* in the kidney, likely in the collecting duct [40, 41]. Collecting duct knockouts of PPAR-*γ* is able to excrete salt loads more easily although there is no end effect on blood pressure on normal salt diets [40, 41]. PPAR-*γ* knockout blocked the effect of TZD on mRNA expression of the sodium channel ENaC-*γ* although the baseline level in the knockout was higher [40].

One recent report regarding the association between rosiglitazone treatment and significantly increased risk in myocardial infarction as well as an increased risk with borderline significance in death from cardiovascular causes has brought a lot of attention to the safety of this drug [34]. The findings were based on limited access to the original data, and meta-analysis used to reach the conclusions is always considered less convincing than a large prospective trial designed to assess the outcome of interest. Such a prospective study is indeed ongoing and the investigators performed an interim analysis and found that rosiglitazone was not significantly associated with increased risk of myocardial infarction and death from cardiovascular causes, although the findings were inconclusive because of the incompleteness of the study [35]. One side effect of rosiglitazone this interim analysis did confirm is the increased incidence of heart failure [35].

Although the findings need to be confirmed, the possible adverse effects of rosiglitazone in myocardial infarction and death from cardiovascular causes are worrisome due to the fact that diabetic patients are already at higher risk for cardiovascular diseases. The mechanisms of the possible adverse effects are uncertain, and could involve myocardial as well as vascular changes. Pioglitazone, another member in the same TZD class, does not seem to have these side effects [119]. In comparison to rosiglitazone, pioglitazone appears to have more beneficial effects on lipid profile [120], which may be one of the contributors to these side effects. However, the exact mechanisms and molecular basis are yet to be explored. In order to ultimately understand this drug and help new drug design, it is critical to address questions such as whether PPAR-*γ* is mediating these effects of rosiglitazone and whether heart (cardiomyocytes, cardiac fibroblasts, endothelial cells, or smooth muscle cells) is the direct target.

The cardiovascular phenotypes of these gain- or loss-of function studies are summarized in Table 1.

## 5. CONCLUSIONS

PPAR-*γ* is now firmly established as an important player in cardiovascular diseases. Understanding the mechanisms of PPAR-*γ* action in heart and vascular cells where action on NF-*κ*B appears to be important in controlling growth and inflammation may lead to improved targeting of the PPAR-*γ* activity in these cells. The interactions of PPAR-*γ* with other nuclear transcription factors which have partially overlapping effects such as the PPAR-*α*, PPAR-*δ*, and LXR will likely reveal a complex control system of inflammatory and growth responses to nutrient signaling.

## Figures and Tables

**Table 1:** Cardiovascular phenotypes in gain and loss of PPAR-γ function.

			CH		MI		BP		CAD		AS	
TZDs	Agonism	Gain of function	${↑}^{*}$	[30–33, 47]	${↑}^{**}$	[34]	↓	[17, 49, 50]			↓	[87–91]
Pro12Ala	Human mutation	Loss of function			↓	[57]	↓	[58]				
Pro467Leu	Human mutation	Loss of function					↑	[59, 60]				
Val290Met	Human mutation	Loss of function					↑	[60]				
Phe388Leu	Human mutation	Loss of function					↑	[61]				
Arg425Cys	Human mutation	Loss of function					↑	[62]				
C161T	Human mutation	Loss of function							↓	[63]		
Pro467Leu	Mouse mutation	Loss of function					↑	[69]				
Leu466Ala	Mouse mutation	Loss of function					↑	[70]				
Generalized KO	Transgenic mouse	Loss of function	↑	[71]			↓	[71]				
Cardiac KO	Transgenic mouse	Loss of function	↑	[47]								
Endothelial KO	Transgenic mouse	Loss of function					→	[75]				
Collecting duct KO	Transgenic mouse	Loss of function					→	[40, 41]				

TZDs: thiazolidinediones; KO: knockout; CH: cardiac hypertrophy; MI: myocardial infarction; BP: blood pressure; CAD: coronary artery disease; AS: atherosclerosis *: in animals only; **: rosiglitazone only Numbers in square brackets are the reference numbers.
